# Progress in Neurology

**Published:** 1903-12-19

**Authors:** 


### Progress in Neurology.

*(Concluded from page* 191.)

**Amyotrophic Lateral Sclerosis** is the subject of an excellent paper by Collins.[6] This somewhat rare disease is characterised by the symptoms of progressive muscular atrophy of the Arau-Duchenne type, complicated with bulbar involvement, plus spastic paresis, particularly of the lower extremities and exaggeration of the tendon jerks all over the body. The morbid process on which it is dependent consists of a decay of the peripheral motor neurons in the ventral horns, the terminal arborisations of the central motor neurons, and of the pyramidal tracts themselves, and atrophy of the column and commissural cells of the gray matter. The degeneration extends centrifugally in the peripheral motor neurons, centrifugally and centripetally in the central motor neurons. The degeneration of the pyramidal tracts may be very slight or marked. Thus in Collins' case the pyramidal tracts were intact, but the antero-lateral tracts (*i.e.* association fibres) were degenerated. The clinical course of such cases is different from that of progressive muscular atrophy; whilst the former rarely lasts more than from two to three years, the latter is much slower, lasting from five to fifteen years. In the former there are no periods of cessation or remission, and the patient is ill from the beginning ; in the latter the disease may cease to progress for a time. Again, in the former disease mental symptoms may occur, *e.g.*, suicidal impulse, attacks of meaningless laughter and crying, and evidences of slight dementia. There is thus in the clinical features of amyotrophic lateral sclerosis much that suggests an active destructive disease, and not merely a decay of nervous elements.

**Muscular Movements.**—In the Croonian Lectures on muscular movements and their representation in the central nervous system, Beevor[7] first discusses the muscles concerned in various movements of the hand. He then passes on to the problem of the curious paralysis in lead poisoning, *i.e.*, when this affects the extensor communis digitorum but not the extensores carpi ; as long as the person keeps his fingers flexed he can extend his hand on the forearm, but if he should wish to extend his fingers his first phalanges remain immovable and his hand is flexed on the forearm with as much energy as the efforts he makes to produce extension of the fingers, and, further, he has no power to extend his wrist as long as he tries to do it with the fingers straight. The explanation lies in the fact than in the movement of extending the wrist with the fingers extended, the extensors of the fingers do all the work themselves until the amount of work done is 3 lb., when the extensores carpi join in and help. Now in lead paralysis the extensores digitorum being paralysed they cannot move at all and so cannot reach the amount of 3 lb. which is required for them to attain before the extensores carpi will come in and help. Hence he cannot extend his wrist with straight fingers, and the resulting movement is flexion of the wrist due to the synergic action of the flexores carpi. In the case of a muscle which passing over two or more joints has two or more actions, then if only one of these actions is desired, other muscles are brought into the movement whose actions are antagonistic to those of the muscles which are not required. In the case of the extensors of the fingers the muscles are extensors of the wrist as well ; hence if one tries to extend the fingers only, there is an associated action of the flexors of the wrist. This explains the first point mentioned above. In his second lecture Beevor first discussed the movements of pronation and supination. In pronation the pronator teres and quadratus contract, with, if there be resistance, the flexor carpi radialis and palmaris longus. Supination is performed by the supinator brevis and biceps. The supinator longus (better termed the brachioradialis) is a flexor of the forearm, but has not the slightest action as a supinator. It may act on extreme pronation and when considerable strength is used. The biceps is both a supinator and a flexor ; when used in supinating, the elbow being kept extended, its action as a flexor is neutralised by a concomitant action of the triceps, and if the triceps is paralysed the biceps cannot act as a supinator without flexing the elbow. In this action it is the outer and inner heads of the triceps which contract, the long head of the triceps being an adductor of the upper arm. In flexion of the elbow-joint the biceps, brachialis anticus, supinator longus, and pronator radiiteres, the brachialis anticus is a pure flexor, the pronator radiiteres and supinator longus are flexor pronators, the biceps is a flexor supinator. In advancing the humerus, the anterior fibres of the deltoid, the clavicular head of the pectoralis major, the biceps, and the coraco-brachialis act. The serratus magnus does not come into action until the humerus has been moved through about 45°. It is the inferior part of the trapezius which fixes the scapula at the commencement of the movement, and if this be paralysed the serratus will not step into the breach. This is parallel to the case of lead paralysis described above. Winging or projection of the scapula is thus produced by paralysis both of the trapezius and serratus ; in the former case the deformity is greatest at the beginning of the movement, in the latter after the humerus has advanced through an angle of 45°. The clavicular fibres of the pectoralis major are in voluntary movement elevators of the humerus, the sternal part depressors. If the humerus be elevated vertically, and the clavicular head be stimulated electrically, it depresses the humerus, but in voluntary movements the clavicular part is not found to act as a depressor. In his third Croonian Lecture Beevor said that the sterno-mastoid muscles are the chief flexors of the head; in addition the platysma myoides, omohyoids, and other depressors of the hyoid, act. In extension of the head the only muscles which contract are the clavicular part of the trapezius, the complexus, and the trachelo-mastoid. He could find no evidence, either in health or disease, that the sterno-mastoids act as extensors of the head when the head is in a position of extreme extension. In dyspnœa the sterno-mastoids act, the head being kept fixed by its extensors. The other extraordinary muscles of respiration are the pectoralis minor, latissimus dorsi, serratus magnus, scaleni, and the claviculo-occipital fibres of the trapezius. The latissimus acts in both expiration and inspiration; in expiration the costal origin from the lower ribs acts to fix the ribs to allow of the external oblique muscle to act with precision. The part played in any movement by the antagonists has long been a disputed question—even from the time of Galen. From observations on man it seems certain that in strong movements against resistance the antagonists are always relaxed; and this agrees with the observations of Sherrington, who found on excitation of the cortical centre for flexion of the elbow, there occurred contraction of the flexors of the elbow, and not merely relaxation but inhibition of the tone of the triceps. In slow, weak movements against no resistance, in man, Demeny says that there is a simultaneous action of the antagonists. A muscle may take part in two different movements: for instance, the biceps may act together with the brachialis anticus, supinator brevis, and pronator teres in flexing the arm, or with the supinator brevis in supinating the arm. It is found that in organic lesions of the central nervous system it may be paralysed for the one movement and not for the other. Similarly the clavicular fibres may be paralysed for elevating the shoulder whilst acting as a bilateral muscle in deep inspiration, and the same thing is true of the latissimus dorsi. It has long been asserted that a muscle may take a principal action in a movement which is diametrically opposed to its usual action; for instance, that the supinator longus may act both as a pronator and a supinator. Beevor cannot find any evidence in support of this statement. There is a definite order in which the muscles for the performance of a movement come into action; they do not all act when only slight effort is required, but a certain increase of work has to be encountered before they all act. For instance, in supinating the wrist, the supinator brevis does the work when only the inertia of the bones has to be overcome, but as soon as resistance over one pound is encountered the biceps is called in to help. Hunter stated that there is no one known muscle in the body which we can throw into action separately; and Hughlings Jackson expressed the same thing by saying that the nervous centres know nothing of muscles, they only know of movements. This can be extended as follows: For every voluntary movement there is a definite number of muscles, usually two or more, which come into action in a definite order which cannot be altered, and the individual members of which cannot be omitted and cannot be supplemented. This combination of muscles—the ultimate or minimal components of a movement—is represented in the central nervous system. It seems probable that the cells of the anterior cornua of the spinal cord are not arranged physiologically for such functional purposes. But stimulation of the skin or posterior roots, the cord being separated from the brain, produces definite reflex co-ordinated movements; so that there is in the cord itself a nervous organisation which can produce co-ordinated movements. The "motor" area of the brain depends on sensory impressions for the power of exciting movements, for Mott and Sherrington found that division of all the posterior spinal roots of the brachial enlargement caused loss of voluntary movement of the hand, though electrical excitation of the cortex still evoked the usual movements. It is found that electrical excitation of the cortex produces co-ordination of synergic movements, for instance, stimulation of the centre for flexion of the fingers and thumb produces also extension of the wrist, exactly as in a voluntary movement; and it is impossible to evoke movements of single muscles only by stimulation. There is thus, apparently, a double system of co-ordinating centres, in the cortex and in the spinal cord, but it is unknown whether the mechanism in the cord used for cortical impulses is identical with that used in reflex movements. In the case of conjugate movements of the eyes the co-ordinating centre is in the medulla, for a lesion of the right sixth nerve nucleus prevents a patient from looking to the right, not only with the right but also with the left eye. This suggests that, *e.g.*, in the voluntary movement of "making a fist" the link between the flexors of the fingers and thumb and the extensors of the wrist is in the spinal cord. But in the case of a complicated movement that has to be learnt, *e.g.*, walking, it seems doubtful whether the co-ordination is transferred from the cortex to the spinal cord as the performance becomes automatic.

[6] Amer. Jour. Med. Sci., June, 1903. [7] Lancet, June 20 and 27, 1903.

